# Supplementary figures and images for: The importance of geometry in the corneal micropocket angiogenesis assay
Source: PLoS Comput Biol. 2018 Mar 9;14(3):e1006049. doi: 10.1371/journal.pcbi.1006049 (PMC5862519; doi:10.1371/journal.pcbi.1006049)

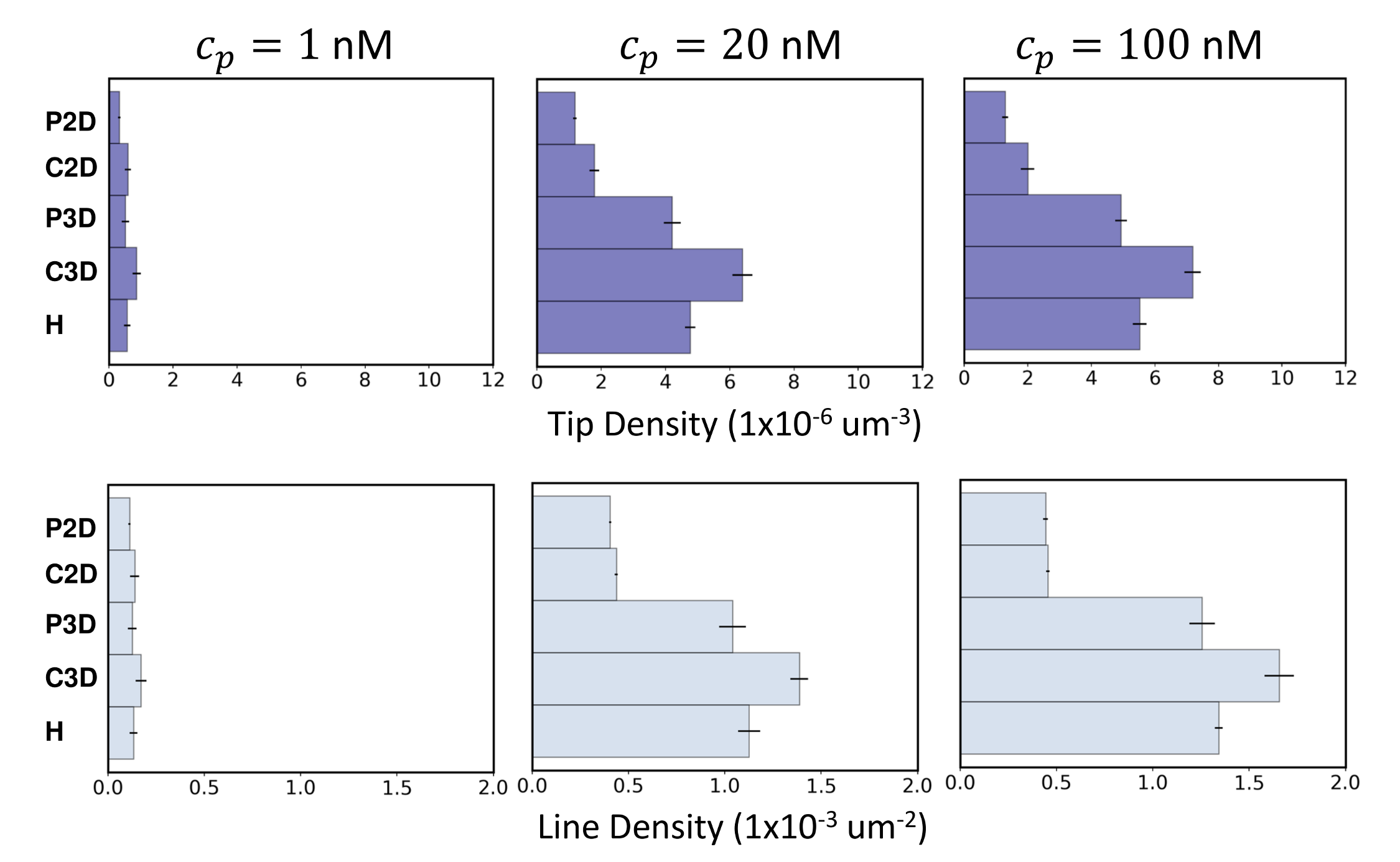

Supplement: S1 Fig — Max tip and line densities after 85 simulated hours for the case with a constant VEGF concentration field. Differences between geometries become more pronounced as the pellet concentration increases. (TIF) [file pcbi.1006049.s001.tif]

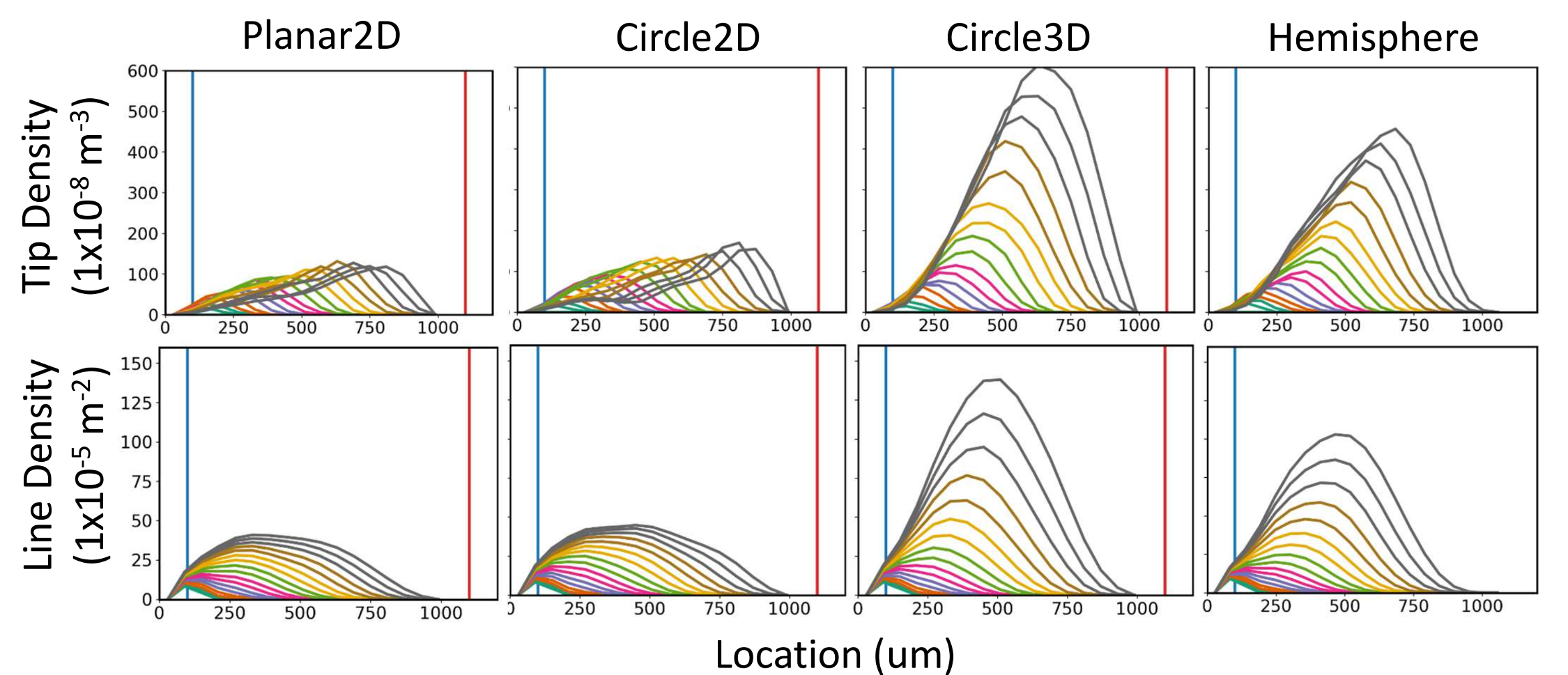

Supplement: S2 Fig — Full tip and line density profiles for a single random realisation in a selection of domains for the case with a constant VEGF concentration field. (TIF) [file pcbi.1006049.s002.tif]
